# Errors of Diagnosis

**Published:** 1875-06

**Authors:** 


### ERRORS OF DIAGNOSIS.

By A. COUVERT, M.D.

I venture to present you the two following cases of error in diagnosis, chiefly for the benefit of our junior brethren. The errors to which I refer were committed by men of mature age and of reputable standing in the profession. This implies a carelessness amounting to criminality, particularly in failing to make a correct diagnosis in so plain a case as that of hydrocele, uncomplicated with hernia or malignant disease of the testicle.

A short time ago I was called on by a gentleman of middle age, from the town of ———, who informed me that two physicians of the place, whom he had consulted, said he had such a disease of the testicle as to require its extirpation. In view of past and prospective services of the cherished member, he declined parting with it on their dicta, and came here for further advice. On inquiry I learned the disease had been gradually advancing for some years. The tumor first appeared around the testicle, and not at the abdominal ring, showing that there was no hernia. There had been no hardness, enlargement or pain of the gland, showing that there was no scirrhus or other malignant disease in it. But, on examination by the eye and the touch, I found some things rather unusual. The whole tumor was elongated, and much like a truncated cone, disproportionately large at the abdominal ring. But I will not consume space by a rehearsal of the appearances and the reasoning that generally make a correct diagnosis so easy in such cases, and which are so clearly set forth by all writers on the subject. Suffice it to say, I plunged in the trocar and canula, and drew off (by subsequent measurement) nineteen ounces of serum, greatly to the surprise and delight of my patient. He preferred occasional tappings to a radical cure, and hence the latter was not attempted.

Many years ago, Mr. M. appeared at my office and made the following statement: He resided near the city of ———. His family physician, a neighbor, concurring with a consulting city physician, condemned one of his testicles to extirpation for malignant disease, and designated a day for the operation. Terrified more and more as the time approached, on the morning of the set day he started in his buggy to consult me, as I had, some years before, treated him for scrofula, with better results than sometimes follow in such cases. I found a simple case of hydrocele, troublesome from its size, with occasional pains in the gland, probably due to his strumous diathesis. The trocar relieved him of eighteen or twenty ounces (by estimate) of serum. He had good health for years thereafter, during which time his wife died and he married again, giving further evidence of his virility, by the subsequent advent of several inheritors of his good name and ample fortune.

Comment is unnecessary. I will only add, nothing but gross ignorance or culpable carelessness could have led to these errors in such cases.

